# Should I Stay or Should I Go (to the Office)?—Effects of Working from Home, Autonomy, and Core Self–Evaluations on Leader Health and Work–Life Balance

**DOI:** 10.3390/ijerph20010006

**Published:** 2022-12-20

**Authors:** Stephanie Maren Neidlinger, Jörg Felfe, Katharina Schübbe

**Affiliations:** Department of Work, Organizational and Business Psychology, Helmut-Schmidt-University Hamburg, 22043 Hamburg, Germany

**Keywords:** health, work–life balance, working from home, remote work, core self–evaluations, autonomy

## Abstract

Leaders represent a high-demand group in organizations. The effects of leaders’ personal and workplace resources on their health and work–life balance have often slipped under the radar, as most studies are directed outwardly and focus on follower outcomes. With this study, we closed a gap in the research and investigated the positive effects of remote work, autonomy, and leaders’ core-self evaluations (CSE) on two important leader outcomes: health and work–life balance. We hypothesized that the relationship between remote work and the outcomes would be moderated by leaders’ CSE and their autonomy—in such a way that leaders with lower resources benefit more from remote work and achieve better health and work–life balance the more days they spend working from home. A sample of 367 leaders reported their frequency of working from home, their autonomy, and CSE. Their health and work–life balance were assessed five months later. Results showed a moderating effect of CSE on both outcomes, indicating that leaders with low CSE benefit more in terms of health and work–life balance. There was no moderating effect of autonomy. Leaders with high resources (autonomy and CSE) had overall higher levels of health and work–life balance regardless of work location. Practitioners in organizations should consider working from home as a resource for leaders, particularly if personal resources are lower.

## 1. Introduction

Since the COVID-19 pandemic forced large parts of the workforce to complete their work-related tasks from home, the concept of remote work has been widely discussed. Recent research has identified opportunities for better health [1,2], and work–life balance [3] if employees are allowed to work from home (WfH).

Working from home increases the opportunities to save time on commuting [4,5], spend more time with families or leisure [5,6], and incorporate more exercise [1,7,8]. Regarding job characteristics, employees working from home experience more autonomy [7,9,10], reduced interruptions, increased productivity, and have even reported higher performance. However, although the overall role of working from home as a resource for employees is widely accepted, it is unknown whether the positive effects also hold for those in leading positions.

Compared to non-leading functions, leadership roles are characterized by unique demands and resources. Regardless of work location, leaders are generally faced with higher and specific demands in terms of decision-making, planning [11], constant availability and interruptions [11], as well as longer work hours [12]—all of which may put leaders’ health and thriving increasingly at risk. Despite changing work mentalities, many leaders still believe it is their duty to arrive at work early, leave work later than their followers, and make sacrifices that impact their working hours [13], which might cause overload and destructive forms of presenteeism [14,15]. Remote leadership may cause additional demands regarding reduced contact and face-to-face communication [7,16]. Motivation, feedback, or tracking task progress may be more challenging from a distance. Nevertheless, working from home may provide specific resources and improve leaders’ overall health and thriving [17].

With the opportunity to work from home, leaders may benefit from increased autonomy, reduced interruptions, and reduced complexity of their environment [7]. Saving time on commute and reducing presenteeism may also increase their work–life balance. We draw on the Job-Demands-Resources (JD-R) model to conceptualize working from home as a job resource for leaders and hypothesize overall positive direct effects of working from home on leader health and work–life balance [18,19]. However, it has not yet been shown whether leaders—with their specific demands—benefit from working from home. As leaders represent an important resource for organizations, knowledge about the effects of working from home on leader health and work–life balance should be prioritized to ensure positive outcomes for the organization as a whole.

Moreover, when examining the effects of remote work on employee outcomes, some inconsistencies emerged [7,20]. Previous studies identified the effects of job and personal resources on who has benefitted more from remote work. Regarding job resources, favorable working conditions such as increased autonomy and decision-latitude led to more positive effects of working from home. In terms of personal resources, the effects of personality, e.g., emotional stability [21] was examined as a moderator of the relationship between remote work and favorable outcomes. Perry and colleagues found that employees were less susceptible to strain when working from home if they had higher emotional stability and more autonomy [21]. Since no study has yet examined a leader sample, it is of interest to examine not only the direct effects of working from home on leader outcomes but also address potential moderators. It is likely that personality as a moderator is even more relevant for leaders than for followers since leadership roles offer more organizational resources, e.g., autonomy and decision-latitude [22].

If organizational resources are better available, as is the case for leaders, individual resources in terms of personality may become increasingly important. This might be especially true when examining the working from home—leader outcome relationship. Personality constructs (e.g., emotional stability and high core self–evaluations) have already been related to coping, resilience, and overall health [21]. Individuals with favorable personality traits possess inherent resources which allow them to adapt to adversities. Even in situations of low external resources, individuals with favorable personality traits show better outcomes since internal resources may be able to compensate for the lack of external resources. However, the opposite might hold true for individuals with low individual resources (e.g., neuroticism and low core self–evaluations). If personal resources are low, external resources such as working from home may be more necessary to achieve positive outcomes.

A personality concept relevant for leaders are core self–evaluations (CSE), which represent fundamental judgments people make about themselves and the world at large [23,24]. The construct combines self-esteem, locus of control, generalized self-efficacy, and (low) neuroticism [25]. High CSE constitute a personal resource and are related to positive health outcomes [26]. Low CSE, on the other hand, represent the absence of a resource: people with low CSE have been found to experience poor health and increased stress and strain.

As we will argue, CSE may be the resource driving whether, and to what extent, leaders benefit from remote work. Individuals with high CSE and thus high personal resources tend to show positive outcomes at home as well as in the office. High CSE may compensate potential risks of a less favourable context. If individual resources are high, successful mastery of demands becomes more likely. If, however, CSE are low, leaders may depend more on a favourable context. Individuals with low CSE might lack resources to compensate and endure adversity. Research has shown that low CSE are related to disadvantages in coping. Therefore, the relationship between remote work and health might be stronger for those lacking CSE because this group may benefit more from a favourable context, like working from home.

This paper aims to close two gaps in research: First, we examined working from home as a resource for leader health and thriving, thus, this represents the first study to focus inwardly on health-related leader (as opposed to follower) outcomes in the context of remote work. This will allow us to shed some light on the conceptualization of working from home as a resource rather than a demand. The second aim of this paper is to enrich the literature on working from home by examining the role of leader personality and shedding light on who benefits from working from home. Additionally, we will examine the role of autonomy for leaders, which may extend existing findings on the positive effects of autonomy in remote work settings.

We recruited a sample of leaders from various occupational backgrounds and used a design with two measurements spaced five months apart to capture both direct and interaction effects of working from home, autonomy, and CSE on leader health and work–life balance.

The study contributes to the literature in multiple ways. First, we focused on a sample of leaders who constitute a high-demand subgroup of the employee population. Leader health and thriving have often slipped under the radar of recent research in favor of the numerically predominant group of employees without leadership functions. Second, we add to the growing body of research on remote work, extending previous studies by adding leader personality as a moderating construct to shed some light on who benefits from remote work. Lastly, we consider the resource-character of both working from home and CSE, highlighting that for leaders lacking the resource of CSE, a compensatory interaction of working from home may increase health and thriving. Examining potential moderators and conditions under which remote work leads to health and thriving is crucial to create a knowledge basis upon which future work and job design will be built. Finally, methodologically, we provide more rigor by using two measurements as compared to most recent studies on remote work, which assessed study constructs within the same measurement. From the results of this study, important implications for both occupational health management and leaders themselves can be derived.

In the following, we will first outline the theoretical background and provide a summary of the relevant literature. Based on the digitalization of work and the specific situation of leaders, we will transfer previous findings regarding working from home to the special situation leaders face in the new and changing world of work. We will elaborate on how working from home as a resource might affect leaders’ health and work–life balance. Additionally, we will examine two further resources on the organizational and personal level: leaders’ autonomy and their core self–evaluations. We will examine the role autonomy, and core self–evaluations play for leaders’ health and work–life balance. Lastly, we elaborate on how each resource might interact with working from home and moderate the relationship between working from home and both leader outcomes.

## 2. Theoretical Background

Scholars and practitioners alike agree that full-time office-based work will not be part of the future. For jobs not requiring office or client-based presence, at least partially remote or hybrid arrangements are believed to become the new normal. The “new world of work” is characterized by the use of telecommunication tools (i.e., video conferences), hybrid and remote work arrangements [7]—all of which are underway to become powerful resources within the organizational context. This development is accompanied by a shift in focus: rather than adapting their lives to fit the requirements of work, new generations of employees are searching to adapt work to fit their lives [25]. The belief that work has changed due to the COVID-19 pandemic has led to increased efforts to understand what has changed, how it impacts employees, and what can be done to improve health and thriving while working productively [7]. Scholars in organizational research agree that working from home can be a valuable resource for employees.

Organizational research usually differentiates between demands and resources to describe the experience and conditions of employees in the workplace. This study is based on the job-demands-resources model (JD-R model) [19], which conceptualizes the interaction between the job demands placed on an individual and the job resources available to cope with those demands. The JD-R model assumes that adverse work outcomes stem from an imbalance between such demands and resources. A job demand is defined as any aspect of work that requires psychological or physiological effort. Resources, on the other hand, include all aspects that help individuals respond to these demands [27]. Job demands and resources can be physical, psychological, social, or organizational in nature. In addition, the JD-R model distinguishes between workplace resources, such as autonomy, and later personal resources [18], such as self-efficacy and optimism [28]. Employee outcomes in terms of health, well-being, motivation, and performance are contingent on resources outweighing demands. If it is the other way around and demands outweigh resources, employees might suffer from psychological distress and poor health. On the other hand, if an individual possesses sufficient resources, the negative influence of job demands on well-being and positive work-related outcomes can be averted [19,27].

**Figure 1:**
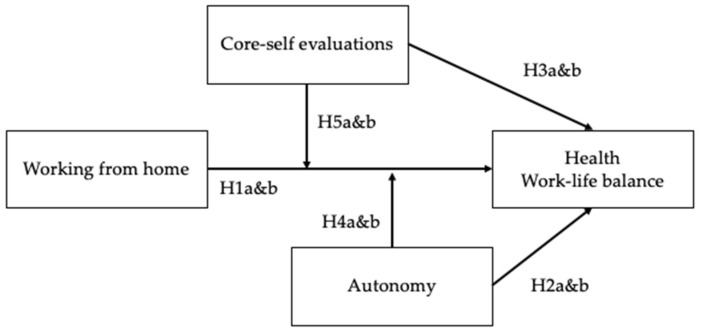
Conceptual model of the moderating effects of autonomy and CSE on the relationship between working from home and outcomes.

According to the taxonomy of the JD-R model, working from home and autonomy constitute organizational and workplace resources, while CSE is a personal resource. The resource potential of autonomy and personality constructs, such as CSE, has previously been established. Working from home has gained popularity in research since the shift in work brought about by the pandemic, and its resource potential needs to be examined further, especially for specific groups, e.g., leaders. Figure 1 illustrates our research model, which hypothesized direct effects of working from home, and direct and indirect effects of autonomy and CSE on leaders’ health and work–life balance. As we will outline in the following sections of this paper, the effect of working from home may depend on autonomy and CSE.

### 2.1. Working from Home and Leaders’ Health and Work–Life Balance

For employees in general, resources and demands relating to the success of working from home and outcomes of working from home have been identified by research. Reducing complexity, saving time on commuting, and reducing interruptions are only a selection of reasons stated by employees which liberate more time for them on days they work from home [1,29]. Numerous studies have found support for the assumption that even some days working from home positively influence employee health [30,31] and work–life balance [29,32,33,34]. Explanations of these positive relationships often named decision-making power, autonomy [32,35], fewer distractions, and more efficient use of their time [35].

However, effects for leaders have not been considered yet. For example, it is unknown if positive effects of working from home on health [1,30,31] and work–life balance [32,33,34] also emerge for leaders or if their benefit depends on further individual or contextual factors.

Employees with leading functions make up at least 5% of the employee population [36]. For Germany, for example, this equals a total of 1.9 million leaders in organizations; in the US, the number of leaders in organizations would be at least 7.9 million. Leaders, although one of the most essential resources for organizations and their success, have often slipped under the radar. Studies are reporting elevated risks for leaders: higher stress and strain [37], high-risk decisions, longer hours [12], and increased pressure for impeccable performance [38]. Besides their professional tasks, leaders are also faced with managerial duties, e.g., they are involved in the hiring processes of new team members, deal with conflicts, and are responsible for people development [37]. Their role, therefore, poses both professional and interpersonal challenges. Especially the interpersonal aspects of leadership have seen a drastic change since the start of the COVID-19 pandemic and the transition towards hybrid and remote work [39,40]. In a recent literature review, Eberl and Drews identified a catalog of additional skills leaders need to master to tackle the changes imposed by shifting the leadership role to a digital setting. Skills they included were agility, openness, and an employee focus [16]. With all these extra demands and challenges, leaders’ health and work–life balance may take a toll: leaders have been found to be very susceptible to strain, and reduced detachment from work [41] and are faced with challenges in balancing their work with their private lives [42]. Especially when the COVID-19 pandemic forced leaders and employees alike to transfer to working from home relatively quickly, research and practice highlighted potential challenges for leaders [43] but also voiced opportunities for additional benefits.

The benefits of working from home found for employees, however, cannot be taken for granted as transferable to leaders, as some specific functions and challenges might emerge for leaders when working from home. Although specific additional demands, such as exerting control in virtual settings [7] and isolation [44], might arise from working at home for leaders, the positive aspects of remote work and the facilitations it has brought about for them have also been highlighted [45]. Especially when additional demands of the leadership role are considered, the potential advantages of working from home may become all the more relevant.

Leaders might have an even greater need for additional resources and the benefits brought about by working from home in order to increase their health and work–life balance [29,30,32,33,34,35]. While it has been shown that employees generally benefit from job and personal resources, this might be especially prominent for leaders in the context of remote work.

Due to their higher workloads, leaders are often under time pressure. Time savings are probably the most apparent and obvious advantage of working from home. First, much time can be saved in terms of commuting, which could add up to multiple hours every workday. When leaders save time on commuting, this reduces time pressure and the risk of strain. Even more important, more time could be spent on health-related activities (i.e., being active, working out, cooking a healthy meal) or other private activities (e.g., hobbies, family). Less commuting also liberates more possibilities to schedule family and work, i.e., read a bedtime story to a young child who would otherwise be asleep after the day’s commute, thus increasing their work–life balance. Moreover, commuting constitutes a stressor itself (traffic, delays, overcrowding, and infection risks in public transport). Reducing these commuting stressors may also increase well-being and health [46].

Leaders, in particular, must manage multiple tasks simultaneously and cater to their followers’ needs, resulting in higher health-related risks. In an interview study, Zimber and colleagues reported that leaders predominantly stated short or no breaks, economic performance pressure, psychological strain, and multiple health-related problems, such as sleep disorders, high blood pressure, and lack of time to spend on health prevention [47]. Additionally, leaders experience frequent interruptions. Interruptions enhance strain because additional time and effort are needed to resume a task after interruptions or when tasks cannot be completed as planned. Studies have also found that working from home reduces interruptions [48]. At home, leaders might be better shielded because there are fewer spontaneous contacts. Accordingly, Kirchner and colleagues reported that leaders find it easier to focus when working from home than employees [45]. They also found that leaders struggled less than employees with deciding which task to do next when they worked from home. This hints at increased productivity for leaders. With fewer interruptions, focus and performance could benefit, thus also saving time, reducing the risk of errors and, therefore, reducing strain. The freedoms and resources provided by working from home could provide crucial relief.

Leaders, in particular, must communicate with others (team members, customers, boards, etc.). In office-based settings and before videoconferencing was established at a larger scale, much time was required to attend in-person meetings or travel for work. With working from home, travel and in-person meetings are reduced. Video-based meetings are often shorter and time for changing meeting rooms or buildings can be saved, thus freeing up time to spend on other tasks or health activities. Leaders’ specific roles and demands make leaders a predestined group for the positive effects of remote work.

Overall, the advantages of working from home may outweigh the potential additional demands for leaders [43]. As we argue, leaders, in particular, should be able to benefit from advantages specific to working from home. We thus postulate that more days spent working from home also acts as a resource for leaders’ health and work–life balance:

**Hypothesis** **1:** *Working from home is positively related to leaders’ health (a), and work–life balance (b).*

### 2.2. Autonomy, Health, and Work–Life Balance

Autonomy describes the extent to which employees experience freedom and independence concerning timing, sequence of completion, and the ability to make decisions at work [49]. Autonomy has been identified and conceptualized as the most important resource for employees [19,27,30,50,51]. Within the job-demands-resources model [19], autonomy is conceptualized as an organizational resource related to the job itself. Autonomy has been found to be critical to employees’ health and well-being [52]—those with higher autonomy can create more opportunities to cope with stressful situations and adversity [50,53].

Autonomy has been linked to better physical [54] and mental health [55] outcomes for employees, regardless of their leadership role. Autonomy has even been related to health-related behavior changes in a meta-analysis with a strong effect size of *g* = 0.81 [56], highlighting the role autonomy at the workplace can have for employees’ health outcomes [9]. Most of these studies, however, have been conducted in traditional, office, or on-site work settings.

Studies on leader autonomy and its effects on leader outcomes remain scarce to non-existent. While leadership styles, such as transformational leadership, can positively affect followers’ perceptions of their job autonomy [57], the effects of leaders’ autonomy on outcomes are still relatively unknown. A recent study by Krick and colleagues reported a significant interaction effect of leaders’ autonomy with their value of their own health on their self-care [58]. However, the effects of autonomy on leader health in remote working contexts remain scarce [59], and it remains unclear whether the effects of autonomy on health and work–life balance emerge for leaders in remote settings.

In general, the amount of autonomy might greatly depend on the work role [50]: in a qualitative study, Grant and colleagues concluded that level and responsibility within the organization were important drivers of autonomy [32]. They reported that those without leading functions reported less autonomy when working remotely than those with managerial roles.

While leaders seem to experience more autonomy in general, the role of autonomy when working from home might be especially beneficial. In remote contexts, leaders could experience more scheduling autonomy because they can choose when to address their followers’ questions and inquiries. Since there is reduced in-person communication, and communication occurs mostly via chat or e-mail, leaders might experience more autonomy in scheduling when to address a question.

Moreover, autonomy helps people to make faster decisions and react flexibly without consulting superiors or asking permission. Waiting for decisions is associated with uncertainty and leads to delays, which can cause stress. This may be more crucial when working from home, where there are fewer spontaneous opportunities to coordinate with superiors at short notice. Autonomy may help accelerate processes and achieve goals faster and more effectively, thus saving time and reducing uncertainty with a potential positive effect on health and work–life balance. Overall, there are numerous positive effects of greater autonomy in remote work settings [9]. Lange and Kayser highlighted the importance of autonomy as a resource in remote work settings, arguing that in the absence of workplace-based resources (i.e., social contacts and colleagues), autonomy may become more important [30]. For work–life balance, the positive effects of autonomy in remote work settings have been established only by a few studies: in a qualitative study, interviewees in 100% remote work arrangements reported higher flexibility and autonomy, which they directly linked to increases in their work–life balance [32].

We expect to replicate and extend previous findings by examining the role of autonomy for health and work–life balance for a leader sample with varying degrees of working from home and postulate:

**Hypothesis** **2:** *Autonomy is positively related to health (a), and work–life balance (b).*

### 2.3. Core Self–Evaluations, Health, and Work–Life Balance

Core self–evaluations (CSE) refer to basic assessments of one’s worth, effectiveness, and general abilities [23] and have been an essential addition to personality literature for the past two decades. CSE are a set of closely related traits which include emotional stability, an internal locus of control, self-esteem, and self-efficacy. Individuals with higher CSE tend to evaluate situations more positively and show higher degrees of motivation. They exhibit confidence in their ability to influence the world around them [24]. However, for individuals with low CSE, studies have observed undesirable workplace outcomes, such as reduced abilities to cope with social stressors [60] or poor psychological health [26]. Judge and colleagues found that the construct of core self–evaluations predicted outcomes better than any of the four characteristics it summarizes [23,61,62].

Resource theories differentiate organizational, job, social and personal resources. High CSE are conceptualized as a personal resource [60,63,64,65]. They reflect a high ability to cope with work adversity or stressors [17]. On the other hand, low CSE represent a lack of personal resources and lowered ability to cope with work adversity or stressors. Especially for leaders, who, compared to employees, are faced with higher demands, the effects of low CSE on health and work–life balance could be detrimental [11,12,66].

Previous studies on leaders’ CSE have mainly focused outwardly on their effects on leadership style and follower outcomes. Doci and colleagues found that high leader CSE were positively related to transformational leadership behaviors [67,68]. High CSE were also found to be positively related to self-leadership [69] and to follower perceptions of servant leadership [70]—the important role of high CSE for displaying positive leader behaviors has thus been established. Positive work-related outcomes for employees, regardless of leadership role, have also been examined in regard to CSE: job satisfaction and work engagement [64], as well as motivation, performance, and goal-setting behavior [61]. In addition to work-related outcomes of high CSE, individual outcomes have also received some attention: multiple studies found that high CSE were related to fewer physical health problems [63,71]. Some studies also found positive effects of CSE on mental health [71]. A study by Grisslich and colleagues showed a moderate relationship between CSE and work–life balance within a sample of university students [72]. For work contexts, however, the results of CSE and work–life balance remain scarce. At the office, regulations might be inhibited by playing a role or the need to interact with colleagues or clients [73]. To sum up, CSE are a well-established concept within research on work and individuals with high CSE benefit from more positive outcomes—both work- and health-related—than individuals lacking CSE as a resource.

However, what has been established thus far might not hold for those in leadership positions facing specific and heightened demands because their work characteristics might be too different from previously examined samples of employees. Leader outcomes as a result of their own CSE are only recently gaining popularity and focus in research studies. Köppe and Schütz found high leader CSE positively related to leaders’ self-care and suggested important implications for health and leader well-being [74]. To our knowledge, no studies linking leaders’ CSE to their health and work–life balance have been conducted thus far. Furthermore, very few empirical studies exist on the implications of CSE in remote working contexts. An editorial by Terry [52] suggested that locus of control, one of the central concepts of CSE, should be examined regarding its effects on the relationship between new work arrangements and work outcomes. Self-efficacy, a second central CSE concept, was found to reduce work-related stress and anxiety, and also led to increased health for employees working remotely [30]. For work–life balance, traditional personality concepts like the Big 5 seem to have no effect [75]. Effects for CSE have only been found for reduced work–family conflicts [76], heightening the importance of examining CSE regarding its predictive power for work–life balance.

Within the context of working from home, leaders can also make use of their core self–evaluations and benefit from their resources. The context of working from home might hold new circumstances and changes, but those with high CSE usually adapt well to any change, learn to craft their environment, and be highly motivated [75]. For example, communication in digital work settings is happening in a less structured, restricted way. Those with high CSE might be equipped with the necessary perseverance and self-efficacy to tackle these challenges. High emotional stability, an internal locus of control, self-esteem, and self-efficacy could prove helpful across many contexts when developing solutions and drawing on their creativity when it comes to solving technical problems. Individuals with low CSE most likely experience more struggles in either work setting, like strong emotional reactions when a video call will not start correctly or helplessness when confronting technical hassles. Moreover, balancing work and private life may be more challenging. We thus postulate:

**Hypothesis** **3:** *Core Self–Evaluations are positively related to health (a), and work–life balance (b).*

### 2.4. Moderating Effects of Autonomy and CSE

Not all leaders have high job (autonomy) and individual (CSE) resources. Considering the specific demands for leaders, those with lower autonomy and lower CSE may even benefit more from working from home. Leaders with high autonomy and high CSE have enough resources at their disposal to cope with the requirements of their job. They may not depend as much on additional resources like working from home and thus experience no additional benefit from it. In the literature, resources have been shown to influence relationships between work characteristics and outcomes indirectly. CSE and autonomy have been shown to exert influence as moderating variables by buffering against job demands and stressors [76], as well as boosting desirable relationships [63].

We draw on the job-demands-resources model [19] to examine the role of autonomy and CSE as moderators of the relationship between working from home and health and work–life balance. According to the JD-R model, low autonomy and low CSE represent the absence of two important resources. We suggest that working from home plays a more significant role as a resource for leaders with low CSE (low personal resources) and low autonomy (low job resources) and that therefore resources moderate the relationships between remote work and health and work–life balance.

When working from home, employees often experience reduced demands in multiple dimensions. Regarding work demands, interruptions, distractions, or pressure [7,35] are usually lower. Individuals with lower resources might benefit more from reduced demands. Leaders with generally lower autonomy have more work-family conflict and increased work-related stress [30]. Lower autonomy usually describes reduced scheduling autonomy, lower decision-making, and method autonomy—in short, leaders with low autonomy have less control over their work environment and work itself. Working from home may somewhat ease their situation because they experience less external control and restrictions when working from home. When working from home, leaders will regain autonomy in terms of their work environment, as their work environment is now their home. New feelings of control and power in terms of their surroundings might help them to invest in health and work–life balance.

**Hypothesis** **4:** *Autonomy moderates the positive relationship between working from home and health (a), and work–life balance (b), such that the relationship is stronger for those with lower autonomy.*

For individuals with high personal resources, i.e., high CSE, and high job resources, i.e., high autonomy, work location most likely does not matter—research has shown multiple times that individuals with high resources adapt and master most kinds of situations and achieve desirable outcomes [17,61,65,77,78,79]. Leaders with lower CSE might be susceptible to problems with their self-management and be preoccupied and hesitant, which could negatively affect their work [80]. For leaders with lower CSE, working from home also holds the potential to bring about important changes to their work. Those with lower personal resources may benefit more from working remotely. Working remotely might bring relief because work can be done at their own pace, and they experience more freedom to effectively cope with their difficulties. For leaders with high CSE, the benefit of working from home is most likely lower: their high CSE help them to cope with most of their work-related challenges—having the ability to do so at the office could translate to working from home.

In support of our reasoning, Das and colleagues found that remote work represented a powerful resource for employees with lower individual resources (i.e., having depression or anxiety) [81]. This group reported more negative experiences in office settings with frequent social interactions and expectations of immediate reaction. When allowed to work from home, they reported increased control, less emotional drainage from socializing, not feeling judged, support from their family at home, more opportunities to craft their ideal environment and listen to their bodies’ needs (i.e., taking a break), thus creating positive implications for their health and work–life balance [81]. When those individuals with low CSE, especially leaders with high role pressures, are allowed to work from home, positive effects on health and work–life balance might emerge:

**Hypothesis** **5:** *Core self–evaluations moderate the positive relationship between working from home and health (a), and work–life balance (b), such that the relationship is stronger for those with lower core self–evaluations.*

## 3. Materials and Methods

### 3.1. Sample and Procedure

To test our hypotheses, a survey study with two measurements was conducted. Inclusion criteria were a current leadership position and working a full-time position. During recruitment, quotas were set so that all levels of working at home (never—5 days per week) were represented by at least 5%. A total of 401 leaders completed both surveys. Role changes (i.e., giving up their leadership function) were reported by 34 leaders between T_1_ and T_2_; those participants were excluded from analyses. The final sample consisted of *N* = 367 German-speaking employees with leadership responsibilities working full-time. Data were collected via online questionnaires. Respondents gave their informed consent.

Participating leaders were, on average, 46 years old (*M* = 46.52, *SD* = 11.95, range 20–68 years), and 62.4% of the sample were male. The majority worked within the private sector (83.7%, public sector 16.3%). The most common branches were public administration (9.3%), logistics, transport, and traffic (9.3%), trade (9.0%), and metal and electronics (9.0%). More than two-thirds of the sample either held a university degree (50.1%) or had completed vocational training (19.3%). Span of control ranged from < 5 employees: (36.2%); 5–10 employees: (24.5%), 10–20 employees (19.6%) to more than 20 employees (19.6%). Participants worked from home on average three days per week (*M* = 2.9, *SD* = 1.55, 7.9% never, 12% one day per week, 18.5% two days per week, 25.6% two days per week, 13.6% four days per week and 22.3% worked exclusively from home). Most participants were married and lived in the same household as their spouse (62.1%). An additional 19.9% lived with a partner but were not married. 3% reported being in a relationship but living in separate households, 3.8% were divorced or widowed, and 11.2% were single and lived alone.

### 3.2. Measures

*Working from home intensity* (WfH). Leaders provided information on their WfH intensity at T_1_, answering to one item based on Gajendran and Harrison [7]: “On average, how many days a week did you work from home in the last four weeks?”. Response options on a six-point scale ranged from 0 (0 days per week) to 5 (5 days per week).

*Autonomy.* Autonomy was assessed at T_1_ using a three-item scale from the Work Design Questionnaire by Stegmann and colleagues [49] and ranked on a five-point scale ranging from 1 (strongly disagree) to 5 (strongly agree). Reliability was *α* = 0.86. An exemplary item was: “I can decide for myself in which order I do my work”.

*Core Self*–*Evaluations*. CSE were assessed at T_1_ and ranked on a five-point scale ranging from 1 (strongly disagree) to 5 (strongly agree). Due to parsimony, a shortened version of the German ACSES-Scale [82] with five items was used. Reliability was *α* = 0.84. An exemplary item was: “I am capable of coping with most of my problems”.

*Health*. Health was assessed at T_2_ using a well-established single item from the German COPSOQ [83] ranked on an 11-point scale from 0 (worst conceivable health) to 10 (best conceivable health). The item was: “If you assign a score of 10 to the best conceivable state of health and a score of 0 to the worst, how many points do you assign to your state of health over the past four weeks?” Assessing health with a single item is an acceptable measurement of the construct [83].

*Work*–*Life Balance*. Work–Life Balance was assessed at T_2_ using an established single item adapted from Hammermann [84]: “My work hours are generally easy to balance with family or social obligations outside of work.” Response options on a five-point scale ranged from 1 (strongly disagree) to 5 (strongly agree). Assessing work–life balance with a single item is an acceptable measurement of the construct [84].

*Control variables*. Due to theoretical reasons, we assessed participants’ age and gender (0 = male, 1 = female, 2 = other) as control variables. No participant within the sample selected “other” as their gender.

We conducted Harman’s one factor test to determine common method variance since data were obtained exclusively via self-report questionnaires [85]. All items used in the study were entered into a principal component factor analysis (without rotation) to test whether variance in the data was accounted for by one single factor. Variance extracted by the single factor was 33.10%, indicating that common method variance was not substantially biasing the data. To assess discriminant validity, we computed the heterotrait-monotrait ratio of correlations (HTMT), which measures similarity between latent variables. Discriminant validity can be assumed when the HTMT is <0.85 [86]. For latent variables in this study, HTMT ranged from 0.16 to 0.36 for all variables, indicating that discriminant validity between our latent variables can be assumed. In terms of convergent validity, we observed the expected relationships between all study variables which are displayed in Table 1: autonomy and core self–evaluations, both conceptualized as resources, showed a moderate significant correlation (*r* = 0.21 **). Similar relationships can be found in the literature [30,52]. Health and work–life balance were also moderately correlated (*r* = 0.30 **), which corresponds to the convergence reported in the literature [1]. 

### 3.3. Analyses

To test all hypotheses, we calculated stepwise regression models including all interaction effects using SPSS version 27. Age and gender were entered into the model as covariates in step one, direct effects of working from home, autonomy, and CSE were entered in step 2, and interaction effects were entered in step 3. To obtain confidence intervals for all interaction effects, we used PROCESS macro version 4.1 in SPSS.

## 4. Results

Descriptive statistics and bivariate correlations between all study variables are presented in Table 1. Intercorrelations between the two outcome variables were moderate. Working from home was positively related to health (*r* = 0.13 *) but not significantly related to work–life balance. Core self–evaluations were positively related to health (*r* = 0.29 **), and work–life balance (*r* = 0.16 **). Autonomy was related to CSE (*r* = 0.21 **). Age was negatively related to working from home intensity (*r* = −0.12 *) and positively related to core self–evaluations (*r* = 0.20 **), autonomy (*r* = 0.21 **), and work–life balance (*r* = 0.11 *). Gender was negatively related to core self–evaluations (*r* = −0.13 *).

### 4.1. Main Effects of Working from Home (H1a and b), Autonomy (H2a and b), and Core Self–Evaluations (H3a and b)

The results of all three moderation analyses are provided in Table 2. The intensity of working from home at T_1_ was related to leaders’ health at T_2_ (*β* = 0.16, *p* = 0.004, *SE* = 0.06). WfH intensity was not related to work–life balance at T_2_ (*β* = 0.05, *p* = 0.096, *SE* = 0.03). Hypothesis 1a was supported, but not Hypothesis 1b.

Leaders’ autonomy was related to health at T_2_ (*β* = 0.24, *p* = 0.031, *SE* = 0.11) and work–life balance at T_2_ (*β* = 0.32, *p* < 0.001, *SE* = 0.06). Hypothesis 2a and 2b were supported. Leaders’ core self–evaluations were related to health at T_2_ (*β* = 0.59, *p* < 0.001, *SE* = 0.10) and to work–life balance at T_2_ (*β* = 0.17, *p* = 0.002, *SE* = 0.06). Hypotheses 3a and 3b were supported.

### 4.2. Moderating Role of Autonomy (H4a and b) and Core Self–Evaluations (H5a and b)

There were no interaction effects of leaders’ working from home intensity and their autonomy on either health or work–life balance. Hypotheses 4a and b were rejected. Additional bootstrapping in PROCESS provided a 95% CI of [–0.28, < 0.01] for the interaction of working from home and autonomy for health and a 95% CI [–0.10, 0.05] for work–life balance. Core self–evaluations moderated the relationship between leaders’ working from home intensity and their health (*β* = –0.23, *p* < 0.05, ΔR^2^ = 0.139), and their work–life balance (*β* = –0.14, *p* < 0.01, ΔR^2^ = 0.130). Hypotheses 5a, and b were supported. Additional bootstrapping in PROCESS provided a 95% CI of [–0.30, –0.06] for the interaction of working from home and CSE for health and a 95% CI [–0.17, –0.03] for work–life balance. Results of all moderation analyses can be found in Table 2.

Since the interaction effect of working from home and autonomy on health was in the expected direction, an additional simple slopes analysis was conducted using PROCESS macro version 4.1 in SPSS. Results showed that for low autonomy, the slope was significant (*β* = 0.42, SE = 0.14, *p* < 0.01) whereas the slope for high autonomy was not significant (*β* = 0.09, SE = 0.12, *p* = 0.44). For those with low autonomy, working from home led to a significant increase in health.

Overall, there were only marginal differences in results from stepwise regression and the results provided by PROCESS.

**Figure 2:**
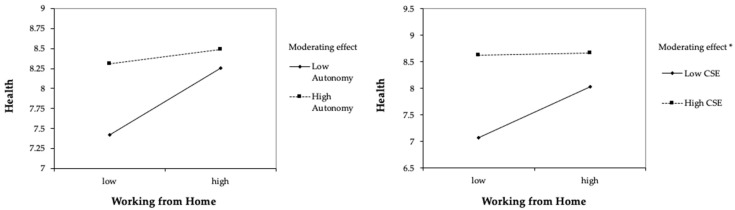
Plot of the interaction between working from home intensity and autonomy (2a), and working from home intensity and core self–evaluations (2b) on health. * *p* < 0.05.

**Figure 3:**
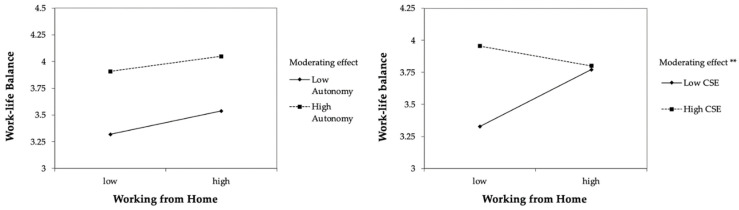
Plot of the interaction between working from home intensity and autonomy (3a), and working from home intensity and core self–evaluations (3b) on work-life balance. ** *p* < 0.01.

Figure 2 and Figure 3 provide graphical representations of all interactions and their effects on health and work–life balance. The figures demonstrate that the positive relationships between leaders’ working from home intensity and both outcomes were stronger for those with low core self–evaluations. Conditional effects for low core self–evaluations were significant for health (*β* = 0.33, *SE* = 0.08, *p* < 0.001) and work–life balance (*β* = 0.14, *SE* = 0.05, *p* = 0.003). For leaders with high core self–evaluations, conditional effects were not significant. Table 3 provides an overview of our findings.

## 5. Discussion

This study aimed to investigate if leaders benefit from working from home and how leaders with low autonomy and low core self–evaluations might benefit from working from home regarding their health and work–life balance. By examining a sample of leaders, we advance the research by investigating the effects of leader personality (CSE) and work characteristics (autonomy) by focusing inwardly, thus, on leader outcomes [41,74,87]. We also add to the growing body of literature examining resources in the context of remote work [7,16,17,17,35].

Consistent with hypotheses on direct effects of remote work, we found that more days spent working from home were related to increased leader health (H1a) after five months. As we suggested, leaders particularly benefit from working from home because they save time in terms of commuting which could reduce strain and pressure, thus freeing up time to spend on health-related activities or with their family. Second, working from home could shield leaders from interruptions and spontaneous contact. Third, work-related travel and in-person meetings are reduced when working from home, thus enabling additional time savings.

We also found support for hypotheses on the direct effects of autonomy and core self–evaluations on leader health (H2a, H3a), as well as on their work–life balance (H2b, H3b). Both results support and extend findings from previous research on health. Associations between CSE (or its four components) and health have been found by multiple papers in different contexts [63,88]. Higher autonomy has also often been related to better health outcomes [30,52,54,63] for diverse samples of employees. Complementing previous research, we were the first to examine this association for a leader sample. As we suggested, high CSE and autonomy represent important personal and job resources. Those with high CSE have confidence in their ability to influence the world around them and higher motivation which can positively influence their health and work–life balance. High autonomy enables leaders to manage their time and schedule work in a way that suits their health and lifestyle also when working from home. For example, autonomy may help make faster decisions, and react flexibly without consulting superiors or asking for permission. This may help to accelerate processes when working from home. Moreover, higher emotional stability, internal locus of control, self-esteem, and self-efficacy (CSE) will help to develop solutions and draw on creativity when it comes to solving technical problems and communication challenges when working from home.

Contrary to our hypothesis, working from home did not increase work–life balance (H1b). We based our theoretical assumptions on evidence in the literature that suggests a positive relationship between remote work and work–life balance. However, there is a body of literature that has discussed problems regarding detachment or conflict with coordination [35] as an implication of remote work: if work loses its location boundaries, leaders who have high workloads could be tempted to work more when they are working from home, thus risking their work–life balance. Leaders have been found to be very susceptible to strain and reduced detachment from work [41] and face challenges in balancing their work with their private lives [42]. Therefore, for leaders, working from home may be ambiguous regarding their work–life balance, and there might be two mechanisms at work. For some employees, the time saved by working from home might enable them to spend more time with their family or manage their private lives, but for others, work boundaries are becoming blurry, resulting in reduced detachment. Further research is needed to examine effects of remote work on leaders’ work–life balance to explain under which circumstances leaders might benefit from working from home in terms of their work–life balance.

We also expanded previous research by investigating if the effect of working from home may depend on CSE and autonomy. Although there was no overall effect for working from home, we found that working from home had a positive effect on work–life balance for leaders with lower CSE (H5b). Also, the positive effect of working from home on health was stronger for leaders with lower CSE (H5a).

This indicates that leaders with low personal resources benefit even more from remote work than leaders with inherently high resources. Working from the comfort of one’s home seems to act as a compensatory mechanism and, to some degree, compensates the absence of personal resources. Leaders with lower CSE might respond more negatively to stress, be susceptible to problems with their self-management, or be preoccupied and hesitant [80]. They might be under intense performance pressure at the office due to interruptions or important decisions which induce stress. When working from home, these stressors might be reduced or attenuated, thus freeing up resources that can be invested toward health and work–life balance. These results are consistent with previous findings from qualitative studies, which suggested that more vulnerable groups might benefit more from remote work [81]. Results found in previous studies not only seem to transfer to leaders, but personal resources in interaction with working from home seem to hold great potential for increasing leader health and work–life balance.

For autonomy, we did not find significant interaction effects with working from home predicting health (H4a) or work–life balance (H4b). When examining the interaction plots in Figure 2a and Figure 3a, although not statistically significant, the courses of slopes for low autonomy are upward. This indicates that the direction of effects shown by our data was in line with our hypotheses. Results of statistical analyses were not significant. However, when examining the slopes separately, we found that for those with low autonomy, working from home increased health, whereas there was no effect for those with high autonomy, indicating that leaders with lower autonomy may benefit more from working from home in terms of their health.

For our hypothesis on leaders’ work–life balance (H4b), autonomy neither increased nor decreased the beneficial effects of working from home. Regardless of their autonomy, leaders benefit from remote work in the same way in terms of their work–life balance. However, the direct effect of working from home on leaders’ work–life balance (H1b) was not significant. This may cause any compensatory effect of working from home for low autonomy not to be as prominent and clear. As we suggested, there may be different underlying mechanisms, which may explain why a moderation effect may not appear. Working from home and an interaction with autonomy might thus be more complex and require further research. Finally, interaction effects were stronger for CSE than for autonomy—one possible reason might be that CSE are a broader resource, while autonomy is more specific.

### 5.1. Implications for Theory and Practice

The findings of this study support the JD-R theory [18,19,27] by highlighting the role of CSE as a personal resource and working from home as a job resource. Similarly, the results align with a theory by Baltes and Baltes, which provides insights into how individuals maintain and replace resources [76]. The selective optimization with compensation (SOC) theory postulates that, facing resource loss, individuals reorganize and select situations and circumstances in which their remaining resources can be optimized [76]. A lack of personal resources might thus be compensated by working from home, a job resource. It would be interesting for future studies to examine whether individuals with low personal resources and the ability to choose how many days per week they work from home recognize beneficial effects of remote work and strategically use working from home as a resource.

We also provide initial evidence for the effects of remote work on leader outcomes, an important sub-population within the workforce that has often been neglected. Due to their specific demands, leaders face different circumstances than followers [89]. We enrich previous research on employee health and the effects of remote work by demonstrating positive effects of working from home on leader outcomes. With our research, we have been able to show that working from home has an independent positive effect on leaders’ health beyond autonomy and CSE. Further theoretical and empirical work is needed to explore additional resources which are beneficial for leader outcomes in the context of remote work.

Several practical implications also emerge from our findings. Substantial literature exists on interventions for leaders—however, the focus of these interventions has often been on the topics of skills, i.e., leadership style or using software. To our knowledge, no published intervention programs have focused on leaders’ personal resources. For leaders with low core self–evaluations, interventions to target their personal resources might be especially useful: our results showed significant increases in leaders’ health and work–life balance due to more days spent working from home for those with low CSE. However, even with fully remote working arrangements, health and work–life balance of those with low CSE were still lower than those of leaders with high CSE as illustrated in Figure 2b and Figure 3b. Targeted intervention designs [90,91] might be a good step in assisting leaders with low CSE to catch up with their high CSE counterparts regarding positive outcomes.

The results of this study, however, raise questions about matters of organizational equality. When designing work and job roles, personality cannot be used as an argument to offer remote work to only a sub-group of leaders with low CSE, as this would undermine equality within the organization. Instead, offering flexibility in terms of location for all leaders, those with low CSE would be enabled to self-select their ideal work locations via resource crafting [92]. Bruning and Champion [92] conceptualized job crafting as encompassing two facets, role crafting, and resource crafting. If organizations granted work location flexibility, those with low personal resources might be enabled to resource-craft and choose to work from home more often.

### 5.2. Limitations and Avenues for Future Research

When discussing and interpreting the results of this study, some limitations need to be addressed. We obtained self-reported data measures, which might be biased and thus not independent. However, to reduce the risk of potential confounding effects of other variables (e.g., current affective state), we used data from two different times. With the data in this study, we can only approximate hints at causality of our results, i.e., the influence of working conditions on health outcomes. We cannot rule out that the experience of health may also influence the assessment of working conditions and self-concept. We call for more longitudinal approaches when investigating the effects of personality and personal resources on employee outcomes to establish causality better.

Another limitation stems from the short scales we used as part of this study which may limit validity and reliability. Both outcome variables, health, and work–life balance were assessed using a single item. For health, participants were asked to rate their state of overall health using an item for the well-established German COPSOQ [83]—some participants might have considered the state of their mental health when responding, while others might have focused on physical health only. A well-established single item assessing work–life balance asked participants to rate the compatibility of their work with their private lives. There are more detailed measures of work–life balance that also consider conflict and enrichment of work–life balance [93]. However, for both health and work–life balance, single-item measures have been studied and found acceptable and valid [94,95,96]. We call for future studies to consider health and work–life balance in more detail and use multiple-item measures.

We suggested that the time saved commuting, as well as reduced spontaneous personal interactions, could be mechanisms that facilitate and add further explanation to the effects of working from home on leader outcomes. Future studies could address additional moderating effects of commute, time savings, and communication to shed some light on additional mechanisms.

Finally, our two-measurement design was spaced five months apart, which might have affected results for autonomy. CSE, a personality concept, shows great stability over time [62]. Its moderating effect on the relationship between remote work and leaders’ outcomes was most likely unaffected by spacing measurements five months apart. Autonomy, however, has a stronger state component [49]. Measuring autonomy and examining its moderating effect on the relationship of an outcome measured five months later might have ignored state autonomy. Future studies might shed light on this assumption by, i.e., assessing study constructs within a diary format to analyze short-term effects.

## 6. Conclusions

This study was the first to integrate the moderating effects of leaders’ core self–evaluations and their autonomy on the relationship between working from home and their health and work–life balance. For all but one direct effect, findings supported our hypotheses: remote work, high core self–evaluations, and autonomy were all antecedents of leaders’ health and work–life balance. We did not find support for one hypothesis, which assumed a direct effect of working from home as an antecedent of work–life balance. Leaders with low core self–evaluations seem to benefit more from remote work regarding health and work–life balance. We thus found support for our assumption that remote work can act as a compensatory resource for low personal resources. For autonomy, however, we did not find a moderating effect on the relationship between remote work and the investigated leader outcomes. Organizations should especially enable leaders with low CSE to work remotely more often, as their health and work–life balance might otherwise be at risk.

## Figures and Tables

**Table 1:** Descriptive statistics and bivariate correlations between all study variables.

		M (SD)/%	1	2	3	4	5	6	7
1	Age	46.52 (11.95)	—						
2	Gender (0 = male)	0.38 (62.4%)	−0.12 *	—					
3	Working from home intensity (T_1_)	2.92 (1.55)	−0.12 *	0.01	—				
4	Core self–evaluations (T_1_)	3.74 (0.94)	0.20 **	−0.13 *	−0.04	(0.84)			
5	Autonomy (T_1_)	3.93 (0.80)	0.21 **	−0.03	−0.03	0.21 **	(0.86)		
6	Health (T_2_)	8.12 (1.75)	−0.02	−0.07	0.13 *	0.29 **	0.15 **	—	
7	Work-life balance (T_2_)	3.72 (0.97)	0.11 *	0.07	0.06	0.16 **	0.30 **	0.30 **	—

Note: *N* = 367. Cronbach’s *α* in parentheses across the diagonal. When no Cronbach’s *α* is indicated, a single item measure was used. * *p* < 0.05; ** *p* < 0.01. Working from home intensity was ranked on a 6-point scale (never—5 days per week). Core self–evaluations, autonomy, and work–life balance were ranked on a 5-point scale. Health was ranked on a 11-point scale.

**Table 2:** Results of moderation analyses.

		Health		Work-Life Balance	
		*β*	*SE*	*β*	*SE*
Step 1	Age	−0.01	0.01	<0.01	<0.01
	Gender	−0.16 ^†^	0.18	0.18 ^†^	0.10
	*R*^2^	<1.0%		2.0%	
	*F*-value	0.93		3.30 *	
Step 2	WfH	0.17 **	0.06	0.05	0.03
	CSE	0.55 ***	0.10	0.12 *	0.05
	Autonomy	0.24 *	0.11	0.32 ***	0.06
	*R*^2^	11.8%		11.3%	
	*R*^2^ *change*	11.3% ***		9.5% ***	
	*F*-value	9.67 ***		9.20 ***	
Step 3	WfH * CSE	−0.23 *	0.10	−0.14 **	0.05
	WfH * Autonomy	−0.08	0.09	0.02	0.05
	*R*^2^	13.9%		13.0%	
	*R*^2^ *change*	2.1% *		1.7 *	
	*F*-value	8.29 ***		7.66 ***	

Note: *N* = 367. Standardized coefficients reported. ^†^ *p* < 0.10, * *p* < 0.05; ** *p* < 0.01; *** *p* < 0.001.

**Table 3:** Summary of study hypotheses and results.

Hypothesis		Results
H1a	Working from home is positively related to health.	supported
H1b	Working from home is positively related to work–life balance.	rejected
H2a	Autonomy is positively related to health.	supported
H2b	Autonomy is positively related to work–life balance.	supported
H3a	Core Self–Evaluations are positively related to health.	supported
H3b	Core Self –Evaluations are positively related to work–life balance.	supported
H4a	Autonomy moderates the positive relationship between working from home and health, such that the relationship is stronger for those with lower autonomy.	rejected
H4b	Autonomy moderates the positive relationship between working from home and work–life balance, such that the relationship is stronger for those with lower autonomy.	rejected
H5a	Core Self–Evaluations moderate the positive relationship between working from home and health, such that the relationship is stronger for those with lower core self-evaluations.	supported
H5b	Core Self–Evaluations moderate the positive relationship between working from home and work–life balance, such that the relationship is stronger for those with lower core self-evaluations.	supported

## Data Availability

The data are available from the authors upon reasonable request.
